# *In Vivo* Evidence of Increased nNOS Activity in Acute MPTP Neurotoxicity: A Functional Pharmacological MRI Study

**DOI:** 10.1155/2013/964034

**Published:** 2013-08-31

**Authors:** Tiing Yee Siow, Chiao-Chi V. Chen, Nina Wan, Kai-Ping N. Chow, Chen Chang

**Affiliations:** ^1^Institute of Biomedical Sciences, Academia Sinica, 128 Section 2, Academia Road, Nankang, Taipei 11529, Taiwan; ^2^Department of Medical Imaging and Intervention, Chang-Gung Memorial Hospital, Chang-Gung University College of Medicine, Taoyuan 33302, Taiwan; ^3^School of Nursing, Queen's University, Kingston, ON, Canada K7L 3N6; ^4^Department of Microbiology and Immunology, Chang-Gung University, Taoyuan 33302, Taiwan

## Abstract

1-Methyl-4-phenyl-1,2,3,6-tetrahydropyridine (MPTP) is a neurotoxin commonly used to produce an animal model of Parkinson's disease. Previous studies have suggested a critical role for neuronal nitric oxide (NO) synthase- (nNOS-) derived NO in the pathogenesis of MPTP. However, NO activity is difficult to assess *in vivo* due to its extremely short biological half-life, and so *in vivo* evidence of NO involvement in MPTP neurotoxicity remains scarce. In the present study, we utilized flow-sensitive alternating inversion recovery sequences, *in vivo* localized proton magnetic resonance spectroscopy, and diffusion-weighted imaging to, respectively, assess the hemodynamics, metabolism, and cytotoxicity induced by MPTP. The role of NO in MPTP toxicity was clarified further by administering a selective nNOS inhibitor, 7-nitroindazole (7-NI), intraperitoneally to some of the experimental animals prior to MPTP challenge. The transient increase in cerebral blood flow (CBF) in the cortex and striatum induced by systemic injection of MPTP was completely prevented by pretreatment with 7-NI. We provide the first *in vivo* evidence of increased nNOS activity in acute MPTP-induced neurotoxicity. Although the observed CBF change may be independent of the toxicogenesis of MPTP, this transient hyperperfusion state may serve as an early indicator of neuroinflammation.

## 1. Introduction

Parkinson's disease (PD) is a neurodegenerative disorder that is caused by the progressive loss of dopaminergic (DAergic) neurons in the substantia nigra pars compacta (SNpc). The cardinal manifestations of this debilitating disease include muscle rigidity, uncontrolled tremor, and bradykinesia. Much of the insight into PD has come from the animal model, in which the condition is induced by administration of the toxin 1-methyl-4-phenyl-1,2,3,6-tetrahydropyridine (MPTP), which faithfully reproduces the pathological hallmarks of PD. MPTP is initially converted to its toxic metabolic form, 1-methyl-4-phenylpyridinium ion (MPP^+^), *in vivo* by monoamine oxidase- (MAO-)B (MAO-B) [1]. MPP^+^ subsequently accumulates in DAergic neurons through high-affinity dopamine transporters [2]. Once inside neurons, MPP^+^ disrupts oxidative phosphorylation by inhibiting mitochondrial complex I of the electron transport chain [3–8]. It is hypothesized that interference with the cellular respiratory machinery leads to rapid depletion of adenosine triphosphate (ATP) and eventually cell death. However, it appears that complex I activity requires reduction of more than 70% to cause significant energy depletion in nonsynaptic brain mitochondria [9] and an *in vivo* study has shown that MPTP causes only a transient 20% reduction in ATP level in the mouse striatum and midbrain [10]. Together these data argue that ATP deficit is the sole factor underlying MPTP-induced neuron loss.

In addition to the ATP-depletion hypothesis, it has been postulated that increased production of nitric oxide (NO) also contributes to MPTP-induced neurotoxicity [11–15]. The impaired oxidative phosphorylation after administrating MPTP causes activation of *N*-methyl-d-aspartate receptors with subsequent increase in the intracellular Ca^2+^ concentration. This leads to the activation of neuronal NO synthase (nNOS), which is a calmodulin-dependent enzyme [16]. The subsequently produced NO combines with superoxide to form the free radical peroxynitrite [17], which in turn degenerates into a more noxious hydroxyl radical to cause cell injury. Nevertheless, NO activity is difficult to assess *in vivo* due to its extremely short biological half-life of only a few seconds [18]. *In vivo* evidence of NO involvement in MPTP neurotoxicity remains scarce.

As well as playing a part in neuroinflammation, NO is known to play a pivotal role in the regulation of vascular tone [19, 20]. The central effect of NO in hemodynamic homeostasis provides a rationale for the present study, which examined the role of NO in the MPTP-induced neurotoxic cascade by monitoring alterations in CBF.

Over the past few decades, magnetic resonance imaging (MRI) has evolved into a powerful imaging modality that offers functional imaging in addition to anatomical information. Flow-sensitive alternating inversion recovery (FAIR) [21], a commonly used magnetic-resonance-based perfusion imaging technique, utilizes tissue water as an endogenous contrast agent to obtain tissue perfusion information. In addition to FAIR, functional imaging modalities such as diffusion-weighted imaging (DWI) and magnetic resonance spectroscopy (MRS) could provide useful information on cytotoxicity and metabolic changes. The noninvasiveness of these techniques enables repeated *in vivo* measurements with high temporal and spatial resolutions.

Combining these MRI techniques with pharmacological inquires, termed pharmacological MRI (phMRI) [22–24], has provided a platform for investigating drug effects *in vivo*. The present study used phMRI to investigate the acute effects of MPTP on the rodent central nervous system (CNS). FAIR, DWI, and *in vivo* localized proton magnetic resonance spectroscopy (^1^H-MRS) were used to, respectively, assess MPTP-induced hemodynamic perturbations, cytotoxicity, and metabolic changes. To further clarify the role of NO in MPTP toxicity, a selective nNOS inhibitor, 7-nitroindazole (7-NI) [25], was administered intraperitoneally (i.p.) to experimental animals prior to an MPTP challenge.

## 2. Materials and Methods

### 2.1. Animal Preparations

All experimental procedures were approved by the Institute of Animal Care and Utilization Committee at Academia Sinica, Taipei, Taiwan. Male Sprague-Dawley rats (4-5 months old) weighing 450–550 g were anesthetized i.p. with a mixture of urethane (800 mg/kg; Sigma, MO, USA) in normal saline and **α**-chloralose (40 mg/kg, Sigma) in polyethylene glycol (Merck, Darmstadt, Germany). Each rat was placed in the prone position and fitted with a custom-designed head-holder. The rats were set up as described previously [26]. Briefly, one femoral vein was cannulated with PE-50 tubing for drug/test solution administration, and an endotracheal tube (PE-280) was inserted for artificial ventilation with an animal ventilator (Model 683, Harvard Instruments, South Natick, MA, USA). The expiratory CO_2_ concentration, which was monitored with the aid of a capnograph (Normocap 200, Datex, Helsinki, Finland), was maintained at 3.5-4.5% by adjusting the tidal volume and ventilation rate. An intravenous (i.v.) injection of a muscle relaxant, gallamine (Sigma), was used to prevent spontaneous ventilation and movement during the image-acquisition period. The initial dose of gallamine was 12 mgs and the maintenance dosage was 6 mg/h. Body temperature was detected by an optical fiber thermoprobe (Model SFF-5, Luxtron, Santa Clara, CA, USA) connected to a Fluoroptic thermometer (Model 790, Luxtron) and was maintained at 37°C by a ceramic heater (Model TH-8105, Tashin, Taipei, Taiwan) throughout the MRI measurements.

The rats were divided into three groups, with six rats in each group. In the first group, the rats received a single, i.v. injection of MPTP (15 mg/kg, Sigma), while the age-matched control group received an i.v. injection of normal saline. The third group of rats received a single dose of 7-NI (50 mg/kg i.p., Sigma) 30 min prior to the i.v. administration of 15 mg/kg MPTP. As shown previously [27], maximal NOS inhibition in the rat brain is manifested within 30 min following the injection of 7-NI i.p.

### 2.2. MRI Protocols

All magnetic resonance experiments were performed on a 4.7-T Biospec 47/40 spectrometer with an active shielding gradient (5.6 G/cm in 500 *μ*s). A 20 cm birdcage coil was used for radiofrequency (RF) excitation, and a 2 cm diameter surface coil was used for signal reception.

Conventional DWI was employed using a pulsed-gradient spin-echo diffusion method, with a repetition time (TR) of 2000 ms, an echo time (TE) of 59 ms, a gradient pulse duration of 20 ms, a time interval between diffusion gradient pulses of 27 ms, and a *b* value of 1300 s/mm^2^. Images were obtained using a 5 cm field of view (FOV), a slice thickness of 2 mm, a 256 × 128 matrix size that was zero filled to 256 × 256, and a total imaging time of 4 min 17 s. The diffusion-sensitive gradients were applied in the read (*x*) direction before and after the refocusing pulse. Hermite-shaped RF pulses with durations of 3 and 1.86 ms were used for the excitation and refocusing pulses, respectively.

The FAIR experiment was implemented with inversion recovery fast spin-echo (IR-FSE) sequences with and without a slice-selective gradient during an inversion pulse. Slice-selective IR-FSE (ssIR-FSE) and non-slice-selective IR-FSE (nsIR-FSE) images were collected using a TR of 3 s, a TE of 20 ms, and an effective TE of 50 ms with an echo train length of 4, a slice thickness of 2 mm, an FOV of 4 cm, an inversion time (TI) of 1.5 s, and a matrix size of 256 × 128. A slab thickness of 5 mm was inverted for the ssIR-FSE images and a hyperbolic secant pulse was used for inversion with a pulse length of 8 ms. The *T*_1_ was measured from nsIR-FSE with TI values of 0.5, 0.9, 1.1, 1.3, 1.5, and 1.9 s.

A point-resolved spectroscopy (PRESS) sequence was used for localized spectroscopy with the following parameters: 5 × 5 × 5 mm^3^ voxel located at the striatal region, spectral width = 4000 Hz, TR = 2 s, TE = 136 ms, number of average = 256, and total scanning time = 8 min 32 s. Water suppression was achieved by chemical-shift-selective saturation, whereby three consecutive Hermite-shaped RF pulses, each of 15 ms duration, are applied followed by spoiling gradients preceding the PRESS sequence. Spectral assignments of the resonance lines *in vivo *were based on the results from *in vitro* ^1^H-MRS.

### 2.3. Data Analysis

All data were processed using commercially available image-analysis software MRVision (MRVision Co., Menlo Park, CA, USA). The *T*_1_ maps were produced using a nonlinear, three-parameter fitting procedure on a pixel-by-pixel basis. The FAIR images were generated by the subtraction of nsIR-FSE images from their corresponding ssIR-FSE images. The resulting images (Δ*M*) were used to generate CBF maps according to the following:

(1) $$\begin{array}{c} f=\frac{\lambda \cdot \Delta M}{2M_{0}\text{TI}\;\exp\left(-\text{TI}/T_{1}\right)}, \end{array}$$

where *λ* is the tissue-blood partition coefficient (0.9 mL/g) [28], *M*_0_ is the thermal equilibrium magnetization, and *f* is the calculated CBF (expressed as mL/min/100 g of tissue, or mL/min/100 g). The *M*_0_ maps were calculated based on the *T*_1_ maps and nsIR-FSE images using the following:

(2) $$\begin{array}{c} M_{\text{ns}}\left(\text{TI}\right)=M_{0}\left(1-2\exp\left(\frac{\text{TI}}{T_{1}}\right)\right), \end{array}$$

where *M*_ns_ is the magnetization in nonselective inversion.

Two regions of interest (ROIs) were analyzed in all cases: the entire cerebral cortex and the striatum. The average CBF was calculated within each ROI. All results are expressed as mean ± SD values. Student's *t*-test was used for statistical evaluations, with the level of statistical significance set at *P* < 0.05.

## 3. Results

**Figure 1:**
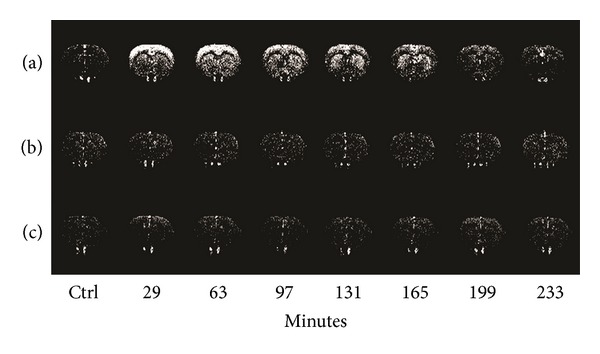
Temporal FAIR images. Representative temporal FAIR images from (a) an MPTP-treated rat, (b) a 7-NI-pretreated and MPTP-treated rat, and (c) a saline-treated (control) rat at baseline (ctrl) and various times postinjection. Progressive elevations of CBF were observed in the cortex and striatum of rats treated with MPTP alone. These elevations were prevented by pretreatment with 7-NI. There were essentially no changes in CBF in either brain region over time in the control rats.

Administration of MPTP did not significantly change either the signal intensity or metabolite concentrations on DWI and *in vivo* ^1^H-MRS, respectively, throughout the 6 h experimental period (data not shown). However, FAIR revealed significant alterations in regional CBF.  Figure 1 shows representative temporal CBF profiles from an MPTP-treated rat, a 7-NI-pretreated and MPTP-treated rat, and a saline-treated rat. The basal CBFs in the cortex and striatum were 109 ± 22 and 102 ± 17 mL/min/100 g, respectively; these values are consistent with those reported previously [21, 29]. There were no significant changes in CBF in brain region over time in the control rats injected with saline alone. However, in rats treated with MPTP alone, there were progressive elevations of CBFs in both the cortex and striatum. In both regions, the changes in CBF became significant at 29 min post-MPTP injection (*P* < 0.05 for the striatum and *P* < 0.001 for the cortex). The CBF was significantly higher in the cortex than in the striatum until 63 min after MPTP injection (*P* < 0.05).

**Figure 2:**
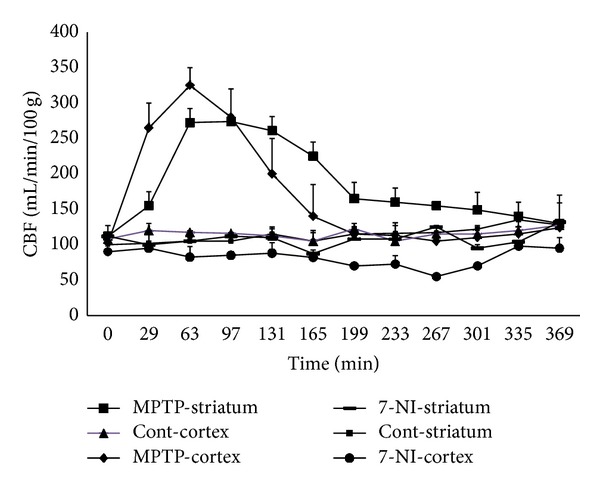
CBF changes over time. Temporal changes in CBF in the cortical and striatal regions of saline-treated (control (Cont)) and MPTP-treated rats. Pretreatment with 7-NI prevented the elevation of CBF induced by MPTP. Data are mean and SD values.

As shown in Figure 2, the cortical CBF of MPTP-injected rats was significantly increased at 29 min after injection (267 ± 51 mL/min/100 g, *P* < 0.001) reached its peak at 63 min (329 ± 27 mL/min/100 g, *P* < 0.001) but subsequently returned gradually to the control level at 165 min (147 ± 51 mL/min/100 g), where it remained until the end of the experiment at 369 min after injection. The CBF increased less in the striatum than in the cortex at 29 min (158 ± 46 mL/min/100 g, *P* < 0.05) and then reached its maximum at 97 min after injection (273 ± 42 mL/min/100 g, *P* < 0.001) but subsequently returned gradually to the control level at 335 min (140 ± 21 mL/min/100 g). The increase in CBF lasted longer in the striatum than in the cortex.

Pretreatment with 7-NI did not change basal striatal CBF, but it significantly attenuated the basal cortical CBF before MPTP treatment (*P* < 0.01). 7-NI completely blocked the MPTP-induced increase in CBF over time in both the cerebral cortex and the striatum. There was no significant MPTP-induced change in CBF in either the cerebral cortex or the striatum throughout the 6 h experimental period following pretreatment with 7-NI.

## 4. Discussion

A possible role of NO in the pathogenic mechanism underlying the actions of MPTP has received considerable attention. There are several lines of evidence that implicated that neuronally derived NO at least partly mediates MPTP-induced SNpc neuronal death. It was previously shown that MPTP neurotoxicity in mice results in an increase in striatal 3-nitrotyrosine (a product of NO and superoxide), which can be attenuated by the administration of 7-NI [30]. In addition, 7-NI can significantly prevent MPTP-related neurotoxicity, as evidenced by the greater number of tyrosine-hydroxylase-immunostained neurons in 7-NI-pretreated mice [15]. Moreover, this protective effect occurred in a dose-dependent manner, indicating that MPTP-induced toxicity is directly proportional to nNOS activity. Further evidence comes from the observation that nNOS-deficient mice are twofold less affected by MPTP than wild-type and heterozygous mice [31]. Together these findings suggest that nNOS-derived NO plays a critical role in the neurotoxicity of MPTP. Although previous studies also suggested that inducible nitric oxide synthase (iNOS) activity was increased after MPTP exposure [31, 32]; due to the relative selectivity of 7-NI, we thus concluded that the transient cerebral hyperperfusion as showed in our study was a result of increased nNOS activity rather than the iNOS.

FAIR is recognized as a completely noninvasive means of visualizing tissue perfusion. Secondary to its noninvasiveness, this technique allows multiple repeated measurements of CBF at sufficiently high temporal and spatial resolutions. Our results obtained using FAIR-phMRI are the first to provide *in vivo* evidence of increased nNOS activity in acute MPTP-induced neurotoxicity. Although FAIR revealed remarkable changes in CBF following MPTP administration, this alteration was not accompanied by metabolic or structural lesions, as evaluated by MRS and DWI. This suggests that FAIR remains a superior tool for detecting early changes in MPTP-induced neurotoxicity.

Apparent diffusion coefficient (ADC) is a DWI-derived quantitative parameter that reflects the degree of tissue water diffusivity restriction. The reduction of ADC has been related to various biological conditions, particularly in the processes that involve cytotoxic edema or increased cellularity (such as inflammation). It has been reported [33] that there was no significant difference of regional ADC values in various brain regions between the PD patients and control group. This is consistent with the result in the present study of rodent PD model. On the other hand, *in vivo* ^1^H-MRS detects low concentration neuronal metabolites to provide surrogate markers for neuronal damage. Previous *in vivo* ^1^H-MRS studies [34–36] showed decreased N-acetylaspartate/creatinine ratio in the lentiform nuclei and striatum of PD patients. However, in the present study, *in vivo* ^1^H-MRS revealed no significant signal change in rat brain after acute MPTP exposure; only CBF change was observed. It could be largely due to the fact that NO is an obligatory regulator of cerebral hemodynamics, where CBF is highly sensitive to the alterations of NO level. Whereas under current dosing regimen, the produced NO level may not be sufficiently high to cause neuronal damage that can be detected by ^1^H-MRS. 

The increase in CBF revealed in our study is probably due to an NO-cyclic guanosine monophosphate (cGMP)-mediated vasodilatory effect [19, 37], which might be independent of the toxicogenesis of MPTP. However, given the strong oxidative power of NO, it is likely that it is at least partly involved in the neurotoxicity of MPTP. The main aim of this study was to demonstrate the spatial-temporal distribution of NO in MPTP-induced toxicity. We believe that our findings will facilitate future studies on the role of NO.

Perfusion neuroimaging studies, either by single-photon emission computed tomography [38] or MRI [39], have generally confirmed the presence of a hypoperfusion state in the gray matter of PD patients. These studies have demonstrated that several cerebral regions, including the posterior parieto-occipital cortex, precuneus, cuneus, and middle frontal gyri, experience decreases in regional perfusion. Paradoxically, the present study revealed a transient hyperperfusion in the cerebral cortex and striatum in the MPTP animal model, which may be related to overproduction of NO. This finding suggests that a similar hyperperfusion state is present in human PD, and this may represent an early neuroinflammation in the brain. Further research should be conducted to examine the existence of this early hemodynamic alteration in human PD.

We found that MPTP injection caused a persistent elevation in regional CBF in the striatum, suggesting that this brain area is a major source of the neurotoxic NO. Consistent with this notion, the striatum contains a rich density of nNOS-positive neurons and fibers [40]. In contrast, there is no evidence for nNOS immunoreactivity in neurons or fibers in the vicinity of the SNpc [40]. Hypothetically, dopamine nerve terminals in the striatum become the primary target for NO, followed by a secondary retrodegeneration of dopamine cell bodies in the SNpc [41]. Consistent with this hypothesis is the observation that MPP^+^ accumulates primarily in the striatal dopamine terminals, but not in SNpc DAergic neuronal cell bodies [42].

While the cerebral cortex exhibits less nNOS activity, there is one possible explanation for the more-prominent increase in the MPTP-induced CBF increase in this region: in addition to the NO-cGMP-mediated direct relaxation of the vascular smooth muscle, the vasodilation effect of NO may also arise from its counteraction to endogenous vasoconstrictors [20]. The observed change in CBF is hence a complex interplay between NO and other vasoactive substances (e.g., angiotensin). Therefore, variations in CBF increases across different brain regions do not necessarily reflect the proportional nNOS activities, since it is likely that there are distinct basal regulation mechanisms in these regions.

It is known that NO plays an important role in the normal regulation of cerebral vascular tone [19]. Our findings also show that 7-NI attenuated the basal cortical CBF prior to MPTP administration. This is consistent with previous reports that 7-NI injection results in a decrease in local CBF in the rat brain [43, 44]. It is worth noting that basal striatal CBF was unaffected by 7-NI injection in the present study, which might have been due to the much lower dosage of 7-NI used.

A major drawback of the present study is the lack of perfusion measurements in the SNpc, which is thought to be a site of PD lesions. This was mainly due to technical limitations associated with the use of the single-slice FAIR technique in this work. Multislice FAIR [45] could be implemented to include measurement of SNpc by increasing the slab thickness of slice-selective inversion such that several slices were contained within it. However, multislice FAIR imaging presents two major problems: (1) the integrity of selective inversion across all slices is questionable (i.e., the imperfect inversion pulse profile across slices causes significant errors) [46], and (2) the multislice FAIR approach introduces an increased transit time delay for those slices farther from the edge of the inversion slice [47]. Together, these limitations hinder accurate measurements using multislice FAIR. Nevertheless, the present results warrant further study of the hemodynamics of the SNpc in acute MPTP toxicity.

It has been reported that rats are less susceptible to systemic MPTP toxicity than mice and primates [48], which might be due to systemic MPTP being extensively metabolized by MAO-B in the rat blood-brain barrier, thereby converting MPTP into MPP^+^ [49]. MPP^+^ is a polar molecule that does not readily cross biological membranes, hence preventing it from reaching sites of injury in sufficient concentrations. This view is supported by the direct infusion of MPTP into the rat SNpc causing a selective 50–70% loss of DAergic neurons, without affecting other neurons or glia at the injection site [50]. Therefore, the differences in susceptibility between species probably arise from their distinct pharmacokinetic profiles. Such differences may have a relatively minimal impact on pharmacodynamic investigations, as in the present research.

As discussed above, several previous studies have shown that 7-NI reduces MPTP-induced neurotoxicity in several animal models, presumably through the inhibition of nNOS. However, Castagnoli et al. demonstrated that 7-NI can also inhibit the MAO-B-catalyzed oxidation of MPTP to MPP^+^ [51]. In sharp contrast, Schulz et al. reported no effect of 7-NI on MAO-B activity [30]. Hence, the exact mechanism underlying the neuroprotective effect of 7-NI against MPTP toxicity remains to be established. Recent data from an *in vitro* study suggested that 7-NI has only a mild MAO-B-inhibitory effect [52]. It is unlikely that such inhibition could affect the interpretation of the results in the present study.

## 5. Conclusion

In summary, this study has demonstrated that systemic administration of MPTP leads to prominent changes in CBF in striatal and cortical regions of the rodent CNS. Such increases can be prevented by pretreatment with the selective nNOS inhibitor, 7-NI. Thus, our results provide the first *in vivo* evidence of NO production in the acute neurotoxicity of MPTP. Given the similarity between the MPTP model and human parkinsonism, this cascade of events may also occur in PD.

## Abbreviations

7-NI: 7-Nitroindazole
ADC: Apparent diffusion coefficient
ATP: Adenosine triphosphate
CBF: Cerebral blood flow
cGMP: Cyclic guanosine monophosphate
CNS: Central nervous system
DAergic: Dopaminergic
DWI: Diffusion-weighted imaging
FAIR: Flow-sensitive alternating inversion recovery
FOV: Field of view
i.p.: Intraperitoneally
IR-FSE: Inversion recovery fast spin-echo
i.v.: Intravenous
MAO: Monoamine oxidase
MPP^+^: 1-Methyl-4-phenylpyridinium ion
MPTP: 1-Methyl-4-phenyl-1,2,3,6-tetrahydropyridine
MRI: Magnetic resonance imaging
MRS: Magnetic resonance spectroscopy
iNOS: Inducible nitric oxide synthase
nNOS: Neuronal nitric oxide synthase
NO: Nitric oxide
nsIR-FSE: Non-slice-selective IR-FSE
PD: Parkinson's disease
phMRI: Pharmacological MRI
PRESS: Point-resolved spectroscopy
RF: Radiofrequency
ROI: Region-of-interest
SD: Standard deviation
SNpc: Substantia nigra pars compacta
ssIR-FSE: Slice-selective IR-FSE
TE: Echo time
TI: Inversion time
TR: Repetition time.

## References

[1] Langston JW, Irwin I, Langston EB, Forno LS (1984). 1-Methyl-4-phenylpyridinium ion (MPP+): identification of a metabolite of MPTP, a toxin selective to the substantia nigra. *Neuroscience Letters*.

[2] Javitch JA, Snyder SH (1984). Uptake of MPP(+) by dopamine neurons explains selectivity of Parkinsonism-inducing neurotoxin, MPTP. *European Journal of Pharmacology*.

[3] Desai VG, Feuers RJ, Hart RW, Ali SF (1996). MPP+-induced neurotoxicity in mouse is age-dependent: evidenced by the selective inhibition of complexes of electron transport. *Brain Research*.

[4] Schulz JB, Henshaw DR, Matthews RT, Beal MF (1995). Coenzyme Q10 and nicotinamide and a free radical spin trap protect against MPTP neurotoxicity. *Experimental Neurology*.

[5] Ramsay RR, Salach JI, Singer TP (1986). Uptake of the neurotoxin 1-methyl-4-phenylpyridine (MPP+) by mitochondria and its relation to the inhibition of the mitochondrial oxidation of NAD+-linked substrates by MPP+. *Biochemical and Biophysical Research Communications*.

[6] Ramsay RR, Dadgar J, Trevor A, Singer TP (1986). Energy-driven uptake of N-methyl-4-phenylpyridine by brain mitochondria mediates the neurotoxicity of MPTP. *Life Sciences*.

[7] Nicklas WJ, Youngster SK, Kindt MV, Heikkila RE (1987). MPTP, MPP+ and mitochondrial function. *Life Sciences*.

[8] Mizuno Y, Sone N, Saitoh T (1987). Effects of 1-methyl-4-phenyl-1,2,3,6-tetrahydropyridine and 1-methyl-4-phenylpyridinium ion on activities of the enzymes in the electron transport system in mouse brain. *Journal of Neurochemistry*.

[9] Davey GP, Clark JB (1996). Threshold effects and control of oxidative phosphorylation in nonsynaptic rat brain mitochondria. *Journal of Neurochemistry*.

[10] Chan P, DeLanney LE, Irwin I, Langston JW, di Monte D (1991). Rapid ATP loss caused by 1-methyl-4-phenyl-1,2,3,6-tetrahydropyridine in mouse brain. *Journal of Neurochemistry*.

[11] Schulz JB, Matthews RT, Jenkins BG (1995). Blockade of neuronal nitric oxide synthase protects against excitotoxicity in vivo. *Journal of Neuroscience*.

[12] Schulz JB, Matthews RT, Klockgether T, Dichgans J, Beal MF (1997). The role of mitochondrial dysfunction and neuronal nitric oxide in animal models of neurodegenerative diseases. *Molecular and Cellular Biochemistry*.

[13] Ara J, Przedborski S, Naini AB (1998). Inactivation of tyrosine hydroxylase by nitration following exposure to peroxynitrite and 1-methyl-4-phenyl-1,2,3,6-tetrahydropyridine (MPTP). *Proceedings of the National Academy of Sciences of the United States of America*.

[14] Spencer Smith T, Swerdlow RH, Davis Parker W, Bennett JP (1994). Reduction of MPP+-induced hydroxyl radica formation and nigrostriatal MPTP toxicity by inhibiting nitric oxide synthase. *NeuroReport*.

[15] Przedborski S, Jackson-Lewis V, Yokoyama R, Shibata T, Dawson VL, Dawson TM (1996). Role of neuronal nitric oxide in 1-methyl-4-phenyl-1,2,3,6-tetrahydropyridine (MPTP)-induced dopaminergic neurotoxicity. *Proceedings of the National Academy of Sciences of the United States of America*.

[16] Garthwaite J, Charles SL, Chess-Williams R (1988). Endothelium-derived relaxing factor release on activation of NMDA receptors suggests role as intercellular messenger in the brain. *Nature*.

[17] Hogg N, Darley-Usmar VM, Wilson MT, Moncada S (1992). Production of hydroxyl radicals from the simultaneous generation of superoxide and nitric oxide. *Biochemical Journal*.

[18] Hakim TS, Sugimori K, Camporesi EM, Andersen G (1996). Half-life of nitric oxide in aqueous solutions with and without haemoglobin. *Physiological Measurement*.

[19] Moncada S, Palmer RMJ, Higgs EA (1991). Nitric oxide: physiology, pathophysiology, and pharmacology. *Pharmacological Reviews*.

[20] Bauer V, Sotníková R (2010). Nitric oxide: the endothelium-derived relaxing factor and its role in endothelial functions. *General Physiology and Biophysics*.

[21] Kim S-G (1995). Quantification of relative cerebral blood flow change by flow-sensitive alternating inversion recovery (FAIR) technique: application to functional mapping. *Magnetic Resonance in Medicine*.

[22] Leslie RA, James MF (2000). Pharmacological magnetic resonance imaging: a new application for functional MRI. *Trends in Pharmacological Sciences*.

[23] Easton N, Marshall FH, Marsden CA, Fone KCF (2009). Mapping the central effects of methylphenidate in the rat using pharmacological MRI BOLD contrast. *Neuropharmacology*.

[24] Martin C, Sibson NR (2008). Pharmacological MRI in animal models: a useful tool for 5-HT research?. *Neuropharmacology*.

[25] Southan GJ, Szabó C (1996). Selective pharmacological inhibition of distinct nitric oxide synthase isoforms. *Biochemical Pharmacology*.

[26] Lee WT, Yin HS, Shen YZ (2002). The mechanisms of neuronal death produced by mitochondrial toxin 3-nitropropionic acid: the roles of N-methyl-D-aspartate glutamate receptors and mitochondrial calcium overload. *Neuroscience*.

[27] Mackenzie GM, Jackson MJ, Jenner P, Marsden CD (1997). Nitric oxide synthase inhibition and MPTP-induced toxicity in the common marmoset. *Synapse*.

[28] Herscovitch P, Raichle ME (1985). What is the correct value for the brain-blood partition coefficient for water?. *Journal of Cerebral Blood Flow and Metabolism*.

[29] Tsekos NV, Zhang F, Merkle H, Nagayama M, Iadecola C, Kim S-G (1998). Quantitative measurements of cerebral blood flow in rats using the FAIR technique: correlation with previous iodoantipyrine autoradiographic studies. *Magnetic Resonance in Medicine*.

[30] Schulz JB, Matthews RT, Muqit MMK, Browne SE, Beal MF (1995). Inhibition of neuronal nitric oxide synthase by 7-nitroindazole protects against MPTP-induced neurotoxicity in mice. *Journal of Neurochemistry*.

[31] Dehmer T, Lindenau J, Haid S, Dichgans J, Schulz JB (2000). Deficiency of inducible nitric oxide synthase protects against MPTP toxicity in vivo. *Journal of Neurochemistry*.

[32] Liberatore GT, Jackson-Lewis V, Vukosavic S (1999). Inducible nitric oxide synthase stimulates dopaminergic neurodegeneration in the MPTP model of Parkinson disease. *Nature Medicine*.

[33] Degirmenci B, Yaman M, Haktanir A, Albayrak R, Acar M, Caliskan G (2007). The effects of levodopa use on diffusion coefficients in various brain regions in Parkinson’s disease. *Neuroscience Letters*.

[34] Federico F, Simone IL, Lucivero V (1997). Proton magnetic resonance spectroscopy in Parkinson’s disease and atypical parkinsonian disorders. *Movement Disorders*.

[35] Federico F, Simone IL, Lucivero V (1997). Proton magnetic resonance spectroscopy in Parkinson’s disease and progressive supranuclear palsy. *Journal of Neurology Neurosurgery and Psychiatry*.

[36] Clarke CE, Lowry M (2001). Systematic review of proton magnetic resonance spectroscopy of the striatum in parkinsonian syndromes. *European Journal of Neurology*.

[37] Murad F (1986). Cyclic guanosine monophosphate as a mediator of vasodilation. *Journal of Clinical Investigation*.

[38] Hsu J-L, Jung T-P, Hsu C-Y (2007). Regional CBF changes in Parkinson’s disease: a correlation with motor dysfunction. *European Journal of Nuclear Medicine and Molecular Imaging*.

[39] Melzer TR, Watts R, MacAskill MR (2011). Arterial spin labelling reveals an abnormal cerebral perfusion pattern in Parkinson’s disease. *Brain*.

[40] Bredt DS, Glatt CE, Hwang PM, Fotuhi M, Dawson TM, Snyder SH (1991). Nitric oxide synthase protein and mRNA are discretely localized in neuronal populations of the mammalian CNS together with NADPH diaphorase. *Neuron*.

[41] Przedborski S, Levivier M, Jiang H (1995). Dose-dependent lesions of the dopaminergic nigrostriatal pathway induced by intrastriatal injection of 6-hydroxydopamine. *Neuroscience*.

[42] Herkenham M, Little MD, Bankiewicz K, Yang S-C, Markey SP, Johannessen JN (1991). Selective retention of MPP+ within the monoaminergic systems of the primate brain following MPTP administration: an in vivo autoradiographic study. *Neuroscience*.

[43] Tanaka K, Fukuuchi Y, Gomi S (1993). Inhibition of nitric oxide synthesis impaires autoregulation of local cerebral blood flow in the rat. *NeuroReport*.

[44] Kelly PAT, Ritchie IM, Arbuthnott GW (1995). Inhibition of neuronal nitric oxide synthase by 7-nitroindazole: effects upon local cerebral blood flow and glucose use in the rat. *Journal of Cerebral Blood Flow and Metabolism*.

[45] Kim S-G, Tsekos NV, Ashe J (1997). Multi-slice perfusion-based functional MRI using the FAIR technique: comparison of CBF and BOLD effects. *NMR in Biomedicine*.

[46] Frank LR, Wong EC, Buxton RB (1997). Slice profile effects in adiabatic inversion: application to multislice perfusion imaging. *Magnetic Resonance in Medicine*.

[47] Calamante F, Thomas DL, Pell GS, Wiersma J, Turner R (1999). Measuring cerebral blood flow using magnetic resonance imaging techniques. *Journal of Cerebral Blood Flow and Metabolism*.

[48] Giovanni A, Sieber B-A, Heikkila RE, Sonsalla PK (1994). Studies on species sensitivity to the dopaminergic neurotoxin 1-methyl-4- phenyl-1,2,3,6-tetrahydropyridine. Part 1: systemic administration. *Journal of Pharmacology and Experimental Therapeutics*.

[49] Kalaria RN, Mitchell MJ, Harik SI (1987). Correlation of 1-methyl-4-phenyl-1,2,3,6-tetrahydropyridine neurotoxicity with blood-brain barrier monoamine oxidase activity. *Proceedings of the National Academy of Sciences of the United States of America*.

[50] Sayre LM, Arora PK, Iacofano LA, Harik SI (1986). Comparative toxicity of MPTP, MPP+ and 3,3-dimethyl-MPDP+ to dopaminergic neurons of the rat substantia nigra. *European Journal of Pharmacology*.

[51] Castagnoli K, Palmer S, Anderson A, Bueters T, Castagnoli N (1997). The neuronal nitric oxide synthase inhibitor 7-nitroindazole also inhibits the monoamine oxidase-B-catalyzed oxidation of 1 -methyl-4-phenyl- 1,2,3,6-tetrahydropyridine. *Chemical Research in Toxicology*.

[52] Thomas B, Saravanan KS, Mohanakumar KP (2008). In vitro and in vivo evidences that antioxidant action contributes to the neuroprotective effects of the neuronal nitric oxide synthase and monoamine oxidase-B inhibitor, 7-nitroindazole. *Neurochemistry International*.

